# DiAlert: a lifestyle education programme aimed at people with a positive family history of type 2 diabetes and overweight, study protocol of a randomised controlled trial

**DOI:** 10.1186/1471-2458-11-751

**Published:** 2011-09-30

**Authors:** Wieke H Heideman, Vera Nierkens, Karien Stronks, Barend JC Middelkoop, Jos WR Twisk, Arnoud P Verhoeff, Maartje de Wit, Frank J Snoek

**Affiliations:** 1 Department of Medical Psychology, The EMGO Institute for Health and Care Research, VU University Medical Centre, Amsterdam, The Netherlands; 2 Department of Public health and Primary Care, Leiden University Medical Centre, Leiden, The Netherlands; 3 Department of Public Health, Academic Medical Centre, University of Amsterdam, The Netherlands; 4 Department of Clinical Epidemiology and Biostatistics, The EMGO Institute for Health and Care Research, VU University Medical Centre, Amsterdam, The Netherlands; 5 Department of Epidemiology, Documentation and Health Promotion, Amsterdam Municipal Health Service, Amsterdam, the Netherlands + Department of Sociology and Anthropology, University of Amsterdam, The Netherlands

## Abstract

**Background:**

Family history is a known risk factor for type 2 diabetes (T2DM), and more so in the presence of overweight. This study aims to develop and evaluate the effectiveness of a new lifestyle education programme 'DiAlert' targeted at 1st degree relatives of people with T2DM and overweight. In view of the high risk for diabetes and cardiovascular disease in immigrants from Turkish origin living in Western Europe, a culturally appropriate Turkish version of DiAlert will be developed and tested.

**Methods/design:**

In this RCT, 268 (134 Dutch and 134 Turkish) overweight 1st degree relatives of patients with T2DM will be allocated to either the intervention or control group (leaflet). The intervention DiAlert aims to promote intrinsic motivation to change lifestyle, and sustain achieved behaviour changes during follow-up. Primary outcome is weight loss. Secondary outcomes include biological, behavioural and psychological indices, along with process indicators. Measurements will take place at baseline and after 3 and 9 months. Changes in outcomes are tested between intervention and control group at 3 months; effects over time are tested within and between both ethnic groups at 3 and 9 months.

**Discussion:**

The DiAlert intervention is expected to be more effective than the control condition in achieving significant weight loss at 3 months, in both Dutch and Turkish Dutch participants.

**Trial registration:**

Netherlands National Trial Register (NTR): NTR2036

## Background

The prevalence of people with type 2 diabetes (T2DM) in the general population is reaching epidemic proportions in many countries, with the total number of people with T2DM projected to rise to 366 million in 2030 worldwide [1]. It is therefore imperative that strategies to prevent the disease are implemented, this might include targeting high-risk populations to increase effectiveness. The increased prevalence is associated with lifestyle dependent risk factors, including being overweight, physical inactivity and unhealthy diet. In addition, the chance of developing T2DM is two to fourfold greater for people with a positive family history (FH) compared to those without, depending on the number of and the distance to the affected family members [2-4]. Since members of families share the same variations of genes, environment and behaviour patterns, family history information could possibly be used for screening and as a vehicle to motivate people at risk for T2DM to change behaviour [5,6]. However, targeting people with a positive family history of T2DM to promote lifestyle changes is an under-explored prevention strategy. In a review [7] we found only three randomised studies in the literature that have reported on the effectiveness of lifestyle-oriented interventions specifically targeted at individuals with a FH of diabetes. Remarkably, none of these trials addressed FH risk information and its implications for education strategies to change lifestyle. Risk information based on family history is of substantial importance in the DiAlert education programme.

Evidence from large prevention studies shows that the risk of developing T2DM can effectively be reduced by almost 60% in people at risk for diabetes as a result of lifestyle changes resulting in sustained modest weight loss[8,9], with persistent benefits over a longer follow-up of at least 10 years [10]. Among the participants in the Diabetes Prevention Program in the US 66% of the male and 71% of the female participants had a first degree relative with diabetes [11] and results from the Finnish Diabetes Prevention Study (DPS) have confirmed the effectiveness of weight loss by lifestyle changes to reduce the risk of diabetes independent of genetic or familial risk of T2DM [12].

To date, the majority of the diabetes prevention studies have evaluated intensive lifestyle and behaviour change interventions, most often including frequent sessions and one-to-one counselling. Translating the evidence to public health is a challenge, and there is a clear need for targeted interventions that balance feasibility and effectiveness to fit primary care and community settings [13]. Evidence from a pragmatic education programme (PREPARE) in the UK showed that 3 hours structured group-based education incorporating a pedometer is an effective strategy for improving glucose tolerance in people at risk, even after 24 months[14,15]. This confirms the effectiveness of short theory based educational interventions aimed at people who are at risk for T2DM, yet not medically ill and unlikely to experience serious symptom distress or functional impairments to prevent T2DM.

Increasing evidence suggests that family history information could contribute to tailored health information, which is potentially more effective in promoting lifestyle changes than health information aimed at 'anyone at risk'[6]. We therefore developed DiAlert, a theory-based lifestyle education program aimed at overweight first-degree relatives of patients with T2DM, to help them reduce their risk of diabetes and related cardiovascular disease. The group-based intervention consists of two sessions of 150 minutes of education with focus on achieving moderate weight loss, by means of improved diet and physical activity.

Here we report on the study design of the DiAlert study and the development and evaluation protocol of the randomised controlled trial (RCT). In this RCT the effectiveness of DiAlert will not only be evaluated in an ethnic Dutch population but in a population from Turkish origin living in the Netherlands as well. Turkish people are an important group of immigrants in our country, according to figures from 2010 383,957 people (2.3%) of the Dutch population is of Turkish origin [16] and they have a higher prevalence of T2DM (5.6%) as compared to the Dutch population (3.1%)[17]. The increased risk of T2DM in this group is believed to be due to familial susceptibility interacting with an unhealthy lifestyle, characterized by high energy and saturated fat intake, along with low levels of physical activity, being overweight and low socio-economic status [18-20]. Few studies have looked at targeted lifestyle interventions in this group and most relate to secondary prevention [21,22]. Comparisons between the two ethnic groups could contribute to the external validity of DiAlert in the future.

### Aims of the trial

The primary aim of this randomised controlled trial is to test the hypothesis that DiAlert can be effectively utilised to promote weight loss in individuals with a first degree relative with T2DM and being overweight at three months, and thereby help reduce their risk of developing diabetes. In addition, we expect to observe significant changes in metabolic, psychological and behavioural parameters 3 and 9 months following the intervention.

## Methods/Design

### Study design

**Figure 1:**
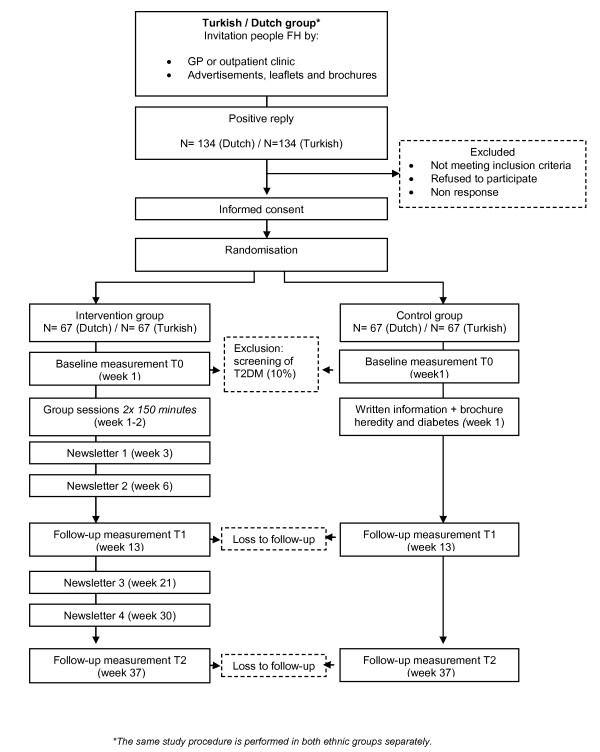
**Participant flow**. A detailed participant flowchart of the DiAlert study

The DiAlert-study is a randomised controlled trial (RCT) testing the effectiveness of a lifestyle education programme in two groups: ethnic Dutch participants and participants of Turkish origin living in the Netherlands. We aim to include 134 Dutch and 134 Turkish overweight first degree relatives of T2DM patients. Measurements are scheduled at baseline, 3 and 9 months after the last group session (see Figure 1).

Information is lacking on the standard deviation of the percentage of weight change over 3 and 9 months of follow-up in a population at risk for T2DM due to family history and overweight. This study is powered to detect a difference of at least 3.5% body weight at 3 months after randomisation (derived from DPP [8]). Assuming a conservative standard deviation of 6%, a power of 80%, and a significance of 0.05 we require a sample size of 50 participants per group at that time point. If during initial screening T2DM is assessed participants will be excluded from the study (10%). Furthermore, we assume an attrition rate of approximately 15%. Therefore we aim to over sample (25%), and include 67 persons in each group (Dutch group: 67/67 Turkish group: 67/67)

### Study population

Eligible are first-degree relatives of T2DM patients (father, mother, brothers or sisters, sons or daughters), between 25 and 65 years of age, and overweight (Body Mass Index of ≥ 25 or waist circumference > 88 cm for females and > 102 cm for males) from ethnic Dutch or Turkish origin.

Exclusion criteria are: people diagnosed with type 1 or 2 diabetes, currently under medical treatment for ischemic heart disease or cancer, diagnosed with a psychiatric disorder, pregnancy or physically/mentally too impaired to participate in the study (e.g. unable to come to the location of the assessments and interventions) and not being able to write and read in Dutch or Turkish.

### Setting

The study population will be recruited in the area of Amsterdam city, the Netherlands, through two strategies: First, GP practices and outpatient clinics will be approached. Patients from GP practices registered with a positive family history and known overweight will be invited by their GP. The second strategy involves an open recruitment strategy through advertisements in local newspapers, leaflets and posters in pharmacies, waiting rooms of GP practices and outpatient clinics. A website with information for participants and professionals to inform people about the study is made available on the internet (http://www.dialert.nl). People can enrol in the study by sending an e-mail or completing a form to be sent by mail. Participation in the DiAlert study is free of charge for all participants. DiAlert group sessions will be delivered in local GP practices and in the outpatient clinic of the VU University Medical Center in Amsterdam facilitated by certified health educators of PRISMA (Pro-active Interdisciplinary Self-Management) [23], who received an additional 4 hours of specific DiAlert training.

### Treatment allocation

After receiving a signed informed consent form from the participants, randomisation will be performed with sealed envelopes. Members of the same family or household participating in the study will be clustered. This will be done to stimulate cohesion in the intervention groups, and to prevent contamination of the intervention effect due to reciprocal communication about the intervention or control condition among family members.

#### Control group

Participants in the control group receive written information about diabetes risk and lifestyle advice to prevent T2DM and a brochure about heredity and diabetes from the Dutch Diabetes Foundation.

#### Intervention group

Participants allocated to the intervention group will be invited to take part in the group-based intervention. Group size is approximately ten participants and the programme consists of two sessions of 150 minutes over two consecutive weeks and 4 newsletters during the next 6 months. For the timing of newsletter mailings see Figure 1 participants flow.

### Intervention

The DiAlert intervention is based on the theory-based diabetes self-management programme PRISMA that was adapted from DESMOND (Diabetes Education for Self-Management in Ongoing and Newly Diagnosed) for the Netherlands [23,24]. DESMOND and PRISMA (hereafter: PRISMA) consist of two interactive group sessions and have shown to be successful at initiating behaviour change in individuals with T2DM [24]. To adapt the program for participants at risk for T2DM we performed a review of existing lifestyle interventions for people with a positive family history of T2DM [7] and consulted different experts.

DiAlert is based on social cognitive behavioural theories, in particular the Health Action Process Approach (HAPA) [25]. HAPA identifies three key determinants of initial change: risk perception, self efficacy and outcome expectancies, leading to intentions and action for health behaviour change. Table 1 provides an overview of the DiAlert modules and aims. In brief, the sessions include explorations of knowledge, impact and concern about T2DM, discussing risk factors for T2DM, insulin resistance and loss of beta cell function, recommendations for balancing energy intake and energy expenditure, goals setting and action planning.

**Table 1:** Outline of the DiAlert programme for first degree relatives of people with type 2 diabetes

Modules	Duration (min)	Sample activity	Aim
**Session 1**	150 min.		

- Introduction	10	General introduction trainer and observers are introduced.	State aims and proceedings of the two group sessions.

- Participant topics	30	All participants are asked about family members with T2DM, and will be encouraged to explore their knowledge, concern and possible impact of T2DM. Motives for participating and burning questions of participants are listed.	Introduction of participants, personal models about T2DM are explored.

- View on personal risk factors	30	Participants share current knowledge of risk factors for T2DM and discuss modifiable and non-modifiable risk factors.	Increase risk perception

	30	*break*	

- How to prevent T2DM?	30	Participants learn about insulin resistance, loss of beta cell function and the positive effects of body weight loss and physical activity.	Increase outcome expectancies for weight loss and physical activity

- Energy balance	10	Advice based on recommendations on nutrition and physical activity. Balancing calorie intake and energy expenditure per day.	Increase outcome expectancies for weight loss and healthy diet

- Homework assignment	10	Self-monitoring diet and physical activity (diary).	Monitoring current lifestyle behaviour

**Session 2**	150 min.		

- Reflections	10	Summary of session 1: Participants reflect on issues raised by the program so far.	Discussing topics of the first session

- Taking control: Nutrition and exercise balance	40	Introduction to calories by comparing different food products. Reading nutrition fact labels.	Knowledge and skills for food choices (calorie and fat intake) to reduce risk factors

	30	*break*	

- Personal action plan	45	Sharing experiences about losing weight. Exploring benefits and barriers for lifestyle change. Participants' stories are used to summarise possibilities/facilities to lose weight. Participants formulate personal action plans to change lifestyle.	Action planning, coping planning and self efficacy for formulated goals

- Burning questions	15	Check whether all questions raised by participants throughout the two group sessions have been answered and understood.	All questions of participants are answered

- Conclusions	10	Summary	Conclusions of the two session are summarised

**Newsletters**			

- 4 Newsletter	4 newsletters are sent by mail with information about health behaviour change following HAPA framework and tips for healthy eating and increasing physical activity.		Focus of the 4 newsletters: 1. outcome expectancies and self efficacy 2. Self efficacy, coping with barriers. 3. Intentions and maintenance 4. Maintenance

Like PRISMA, DiAlert encourages participants to consider their personal risk factors and choose specific goals of behaviour changes by using a non-didactic learning approach. Through eliciting personal stories and respectfully exploring and discussing participants 'personal models' [26] and barriers, the stage is set for reviewing the need for and benefits of behaviour changes, with a focus on healthy food choices and increasing leisure physical activities resulting in a personal action plan to change health behaviour.

A participant manual was developed after investigation of printed education materials of other lifestyle interventions and health promotion leaflets. The manual provides background to sessions and contains information about diabetes prevention, including risk information, tips and tricks to enhance self-efficacy and outcome expectancies for diet and physical activity, resources for the participants such as a table of caloric values and worksheets for the homework assignment and the participants' action plan. In addition, four newsletters will be sent by mail during the follow-up (see Figure 1) providing information on health behaviour change, links to relevant websites, and 'tips and tricks' to stay on track with behavioural changes, physical activity resources in the neighbourhood and recipes for healthy cooking.

To enhance effectiveness and sustainability, participants will be informed about and encouraged to use existing local facilities for healthy lifestyle programs. Participants will be stimulated to seek support after DiAlert from their GP, a dietician or physiotherapist to assist them with their goals to lose weight.

### Pre-testing the intervention

Once the initial draft of the DiAlert intervention was completed, the intervention was pre-tested by a multidisciplinary panel of diabetes professionals, consisting of a registered dietician, a diabetes nurse specialist, two specialist diabetes psychologists, a research assistant, and an expert in the field of diabetes risk communication. The intervention was delivered by a trained psychologist and a health scientist (WH), both certified PRISMA trainers. The feedback from the professionals' panel was overall positive with minor suggestions for further improvement.

Following a framework for development and evaluation of RCTs for complex interventions to improve health (MRC framework) [27] a feasibility and piloting stage was be performed before embarking on a randomised controlled trial. Twenty first degree relatives of T2DM patients were invited to participate in a pilot study to evaluate feasibility, acceptability, participant appreciation, and questionnaire assessments. Based on the evaluation of the pilot the intervention modules were adapted before we set off the RCT.

### Cultural adaptation

After implementation of DiAlert in the ethnic Dutch population, we will assess effectiveness and feasibility of DiAlert in a population of Turkish people who live in the region of Amsterdam in the Netherlands. Cultural adaptation and translations will be performed in order to target DiAlert for this group. By means of a needs assessment general information about the community, demographic data, health status, knowledge of health and cultural related information will be obtained [28]. Possible 'mismatches' in the ethnic Dutch intervention will be identified through literature review, interviews and focus group discussions with lay people from Turkish descent.

Study materials including participant information, newsletters and leaflets will be translated into Turkish with regard to core values, beliefs, norms and other significant aspects of the groups and lifestyles [29].

Questionnaires will be forward-translated by a bilingual health professional with knowledge of both the Dutch and Turkish culture, following guidelines of the WHO for translating instruments [30]. Conceptual rather than literal translations will be taken into account by natural and acceptable language. Backward-translations will be performed by an independent translator whose mother tongue is Turkish. Comparisons between the two translations will be performed and discrepancies will be discussed with both translators.

A pilot test of the culturally targeted intervention will be carried out in a sample of the target population to pre-test study materials and modules of DiAlert. Fidelity and appreciation will be evaluated with questionnaires and interviews.

### Outcome measures

The main outcome measure is change in body weight. Secondary outcome measures include biological, behavioural and psychological outcomes and perceived health status. Predictors for weight loss and health behaviour change (e.g. participant characteristics: gender, initial BMI, SES, number of family members with diabetes, perceived risk and perceived seriousness of diabetes) will be assessed.

### Measurements

Baseline measurements are planned after randomisation and follow-up measurements are planned at 3 and 9 months (see Figure 1 for a detailed flowchart). Baseline and follow-up measurements include physical measures, laboratory tests and questionnaires.

#### Physical measures

The anthropometric assessment will be performed in the GP practice or outpatient clinic by a trained research assistant. Calibrated scales will be used to measure body weight to the nearest 0.5 kg, wearing light indoor clothing and no shoes. Height in cm will be measured to the nearest 0.1 cm on bare feet. Waist circumference will be measured twice with a tape to the nearest 0.1 cm at the level midway between the lowest rib margin and the iliac crest.

Systolic and diastolic blood pressure (in mmHg) will be measured in a seated position with a fully automated blood pressure monitor (OMRON M5-I). All measurements will be performed twice, mean values of the two measurements will be computed.

#### Laboratory tests

Fasting blood samples will be drawn at the laboratory to determine HbA1c, total cholesterol, LDL and HDL-cholesterol, triglycerides, glucose and insulin, to calculate HOMA [31].

#### Questionnaires

All participants will be asked to fill out a questionnaire (20 minutes), either at home via the internet or on paper. The questionnaire consists of cross-cultural validated questionnaires when possible.

#### Participant characteristics

At baseline, socio-demographic data (marital status, highest level of completed education, current employment), reasons to participate in the study, complete family history of diabetes in first and second degree relatives, illness and co-morbidity in the past, and use of medication will be assessed. Ethnicity will be assessed by asking own and parents' country of birth, duration of living in the Netherlands and self-reported ethnicity.

#### Body weight

Questions about body weight include: a) weight loss history (number of attempts to lose weight, received weight counselling by a GP, dietician or physiotherapist during the past 3 months, desired body weight and body weight history over the past five years), b) body weight perception (description of own body weight: answer categories include 'thin', 'average', 'somewhat overweight' and 'overweight') and c) Importance of body weight, assessed by a 5-point likert scale from very important to totally not important.

#### Lifestyle behaviours

Frequency and amount of fruit, vegetable and snack intake per week and nutrition habits will be assessed with a modified version of a frequently used food frequency questionnaire [32]. Moderate, vigorous physical activity and the amount of walking per week will be assessed with the IPAQ short form [33]. Smoking behaviour will be assessed by asking participants if they are a current smoker, an ex-smoker, or a never smoker. In case of a current smoker the number of cigarettes or other tobacco products will be assessed. Alcohol intake on weekdays and weekend days will be assessed separately.

#### Health status

Health outcome will be measured using the EQ5D questionnaire [34]. The Kessler-10 (K10) [35] will be used to assess level of psychological distress (e.g. anxiety and depressive symptoms). The Dutch version of the K10 is appropriate for screening depressive disorders in primary care [36]. In addition, life-events occurred in the past 6 months, and psychological treatment in the past will be assessed.

#### Determinants of health behaviour change

In the HAPA framework determinants of initial change have been identified: perceived risk, self-efficacy, outcome expectancies, leading to intentions and action planning.

Questions about *risk perception *will include perceived causal beliefs, consequences and control of diabetes, which were derived from the revised Illness Perception Questionnaire (IPQ-R) [37]. In addition, comparative risk (a lot lower to a lot higher), estimation of risk (very low to very high) and emotional representation (totally not worried to very worried) will be assessed with 7-point likert scales (questions derived from Claassen et al.[38]). Finally, participants will be asked to score whether they think that lowering the chance of diabetes is: 'very important to totally not important' and 'very easy to very difficult'.

*Self-efficacy *for healthy eating and physical activity is assessed by ten questions using a 4-point likert scale (very uncertain to very certain) [39].

*Outcome expectancies *for a healthy diet and increasing physical activity will be measured with 8 questions with a 5-point likert scale (totally disagree to totally agree) [40]

*Intention *and *action planning *to change health behaviours will be assessed on a 5-point likert scale ranging from totally disagree to totally agree, asking participants whether they plan to consciously eat healthier/exercise more/lose weight and if they have formulated a detailed action plan (what, when, how) for changing diet and physical activity [40].

### Process evaluation

To monitor program implementation a process evaluation will be carried out following a structured process evaluation plan [41]. Fidelity, reach, dose delivered and received of the intervention and materials will be assessed by means of a short questionnaire at the end of the second session. In addition, the health facilitators will be asked to evaluate the group sessions, directly after each session.

### Statistical analysis

Descriptive statistics will be applied to describe the study population at baseline. To determine the effect of the intervention on weight loss and to follow individual change over time we will use generalized linear mixed models and take into account different settings (e.g. intervention groups, trainers), clustering of family members and correlation between observations from same subject; i.e. using a three level structure. Potential confounders and effect modifiers (e.g. BMI at baseline, SES, gender and age) will be investigated. To test predictors of weight loss multiple regression analysis techniques will be performed. The level of significance is set at p < 0.05.

### Ethical approval

The study protocol, information letters and informed consent form were approved by the Medical Ethics Review Committee of the VU University Medical Center.

## Discussion

This randomised controlled trial is designed to evaluate the effectiveness of DiAlert in assisting overweight individuals with a family history of T2DM to lose weight in order to reduce their risk of developing T2DM. There is growing evidence for prevention of T2DM by lifestyle. However, an approach with a focus on family history combined with structured lifestyle education has not been utilized before. We assume that the DiAlert intervention will prove to be more effective than the control condition in achieving significant body weight loss at 3 months and 9 months. In addition, we expect to observe significant changes in metabolic, psychological and behavioural parameters following the intervention in both ethnic groups, resulting in reduced risk of developing T2DM.

The study has some limitations that should be mentioned. First, blinding of the participants will be impossible because they receive the intervention. To limit this bias, participants will not be informed about the outcomes of measurements, except when outcomes suggest diagnosis of diabetes based on guidelines. Furthermore, family history and overweight is not consistently recorded in patient registers of the GP in the Netherlands [42], which might hinder recruitment of participants. Therefore we will utilise different recruitment strategies. By recording the recruitment path of each participant, we will be able to take into account bias due to different motivations of participation in the study.

Despite the growing knowledge in the field of primary prevention, there is an urgent need for well-designed translational studies in populations at high-risk for diabetes. The challenge in offering an intervention in a primary care setting is to find the right balance between efficacy (intensity, follow-ups) and feasibility. Particularly, in the case of people who are just at risk for T2DM and not yet medically ill and not meeting with disadvantages and complications of the disease. To increase feasibility, the intervention will be delivered in close proximity to participants' homes, which could reduce barriers for participation. In addition, because DiAlert consists only of two interactive group sessions, we assume low drop-out rates. At last, delivery in GP practices or in the outpatient clinic could contribute to the perception of the reliability of the given information.

A group-based lifestyle education is a practical method for evidence-based prevention of T2DM in real-life settings [43]. However, the group composition is important to consider, particularly with respect to mix of socio-demographics, health profile (previous health warnings, overweight) and cultural background. By targeting at a population of Turkish origin we will be able to reach a broader population at risk for diabetes in the Netherlands. However, we should take cultural competence of materials and facilitators into account.

### Future implementation

This short, but comprehensive intervention could contribute to the knowledge of prevention of T2DM in public health. If DiAlert proves to be effective in reduction of body weight, implementation will be considered. The process evaluation will provide us with barriers and facilitators that can be used to determine the optimal implementation strategy.

## Competing interests

The authors declare that they have no competing interests.

## Authors' contributions

WH coordinates the study constructed the design and drafted the manuscript. BM, VN, KS, AV participated in the design of the study and revised the manuscript. JT helped to develop the statistical analyses and reviewed the manuscript. MdW revised the manuscript. FS developed the study, constructed the design and revised the manuscript. All authors read and approved the final manuscript.

## Pre-publication history

The pre-publication history for this paper can be accessed here:

http://www.biomedcentral.com/1471-2458/11/751/prepub
